# Detection of 46, XY Disorder of Sex Development (DSD) Based on Plasma Cell-Free DNA and Targeted Next-Generation Sequencing

**DOI:** 10.3390/genes12121890

**Published:** 2021-11-25

**Authors:** Luigia De Falco, Carmelo Piscopo, Rossana D’Angelo, Eloisa Evangelista, Teresa Suero, Roberto Sirica, Raffaella Ruggiero, Giovanni Savarese, Antonella Di Carlo, Giulia Furino, Ciro Scarpato, Antonio Fico

**Affiliations:** 1 AMES, Centro Polidiagnostico Strumentale, 80013 Naples, Italy; rossanad83@icloud.com (R.D.); eloeva@hotmail.it (E.E.); teresasuero@alice.it (T.S.); roberto.sirica@centroames.it (R.S.); raffaella.ruggiero@centroames.it (R.R.); giovanni.savarese@centroames.it (G.S.); dicarloantonella@hotmail.it (A.D.C.); giuliafurino2@gmail.com (G.F.); centroames@libero.it (A.F.); 2 Fondazione Genetica per la Vita Onlus, Via Cuma, 80132 Naples, Italy; 3 Medical and Laboratory Genetic Unit, A. Cardarelli Hospital, 80131 Naples, Italy; carmelo.piscopo@aocardarelli.it; 4 Ambulatorio Medicina Prenatale, PO S. Giuliano, 80014 Naples, Italy; dott.ciroscarpato@gmail.com

**Keywords:** 17β-hydroxysteroid dehydrogenase deficiency, *HSD17B3* gene, non-invasive prenatal testing (NIPT), whole exome sequencing (WES), sex discordance

## Abstract

Mutations in the *HSD17B3* gene cause *HSD17B3* deficiency and result in 46, XY Disorders of Sex Development (46, XY DSD). The diagnosis of 46, XY DSD is very challenging and not rarely is confirmed only at older ages, when an affected XY female presents with primary amenorrhea or develops progressive virilization. The patient described in this paper represents a case of discrepancies between non-invasive prenatal testing (NIPT) and ultrasound based fetal sex determination detected during prenatal screening. Exome sequencing was performed on the cell free fetal DNA (cffDNA), amniotic fluid, and the parents. Libraries were generated according to the manufacturer’s protocols using TruSight One Kits (Illumina Inc., San Diego, CA, USA). Sequencing was carried out on NEXT Seq 500 (Illumina) to mean sequencing depth of at least 100×. A panel of sexual disease genes was used in order to search for a causative variant. The finding of a mutation (c.645 A>T, p.Glu215Asp) in *HSD17B3* gene in amniotic fluid as well as in cffDNA and both parents supported the hypothesis of the *HSD17B3* deficiency. In conclusion, we used clinical exome sequencing and non-invasive prenatal detection, providing a solution for NIPT of a single-gene disorder. Early genetic diagnoses are useful for patients and clinicians, contribute to clinical knowledge of DSD, and are invaluable for genetic counseling of couples contemplating future pregnancies.

## 1. Introduction

Deficiency of 17β-hydroxysteroid dehydrogenase type 3 (17β-HSD3) is a rare autosomal recessive 46, XY disorder of sex development (DSD) [1,2]. *HSD17B3* isoenzyme is present almost exclusively in the testes and converts Delta 4-androstenedione (D4) to testosterone [3,4]. To date several missenses, splice junction, and frame shift mutations in the *HSD17B3* gene have been reported thatcause *HSD17B3* deficiency [5]. Affected individuals have testes often found in the inguinal canal or in a bifid scrotum/labia majora [1,2,3] and normally developed Wolffian duct derivatives, but show undervirilization of the external genitalia, which often appear female with clitoromegaly, a blind-ending vagina, and at times, labial fusion [1,2,3]. They are usually raised as females [4], and virilization tends to occur at puberty due to peripheral conversion of androstenedione to testosterone by other 17ß-HSD isoenzymes [6]. Since its introduction into clinical practice in 2011, non-invasive prenatal testing (NIPT), which uses massively parallel sequencing analysis of cell-free fetal DNA (cffDNA) in maternal plasma, is widely used to detect the most common autosomal including trisomy 21 (T21), trisomy 13 (T13), trisomy 18 (T18), and sex chromosome aneuploidies [7,8,9]. While this technology has been widely applied as a highly sensitive screening test for aneuploidy, there has been relatively little clinical application for the diagnosis of monogenic disorders [10]. Studies reporting dominant de novo mutations or dominant mutations of paternal origin from cffDNA have been previously described [11,12]. For recessive disorders where the patient is a compound heterozygote, the same technology can be applied, because the mutations in both parents are different [13]. However, for maternally inherited variants, this is much more challenging and relies on dosage-based techniques to detect small differences in the levels of mutant and wild-type alleles [14,15]. The patient described in this paper represents a case of discrepancy between NIPT and ultrasound based fetal sex determination detected during prenatal screening. We used targeted sequencing and non-invasive prenatal detection providing a one-step solution for NIPT of a single-gene disorder.

## 2. Materials and Methods

### 2.1. Clinical Report

The pregnant woman we describe is a Caucasian female of 25 years old and was at 12 + 2 weeks of gestation when NIPT was performed. Family history of both the pregnant woman and her partner was negative for genetic or chromosomal diseases. Additionally, they did not report consanguinity, but, during the genetic counselling, the investigation of both family trees allowed them to understand that they came from the same geographic area. At 14 weeks of gestation, a routine ultrasound scan was performed to measure nuchal translucency (NT) and to evaluate fetal anatomy, but imaging of genitalia was not possible during this examination. Again, a routine fetal morphology assessment at 18 weeks showed no structural anomalies, and female external genitalia was identified. Because a discordance occurred between NIPT results and ultrasound sex determination, a second cffDNA was performed at 20 weeks of gestation. Results from NIPT and ultrasound suggested a need for prenatal diagnosis. The pregnant woman underwent amniocentesis, and 15 mL amniotic fluid was retrieved for QF-PCR and GTG banding to confirm the NIPT results.

### 2.2. DNA Extraction, cffDNA Isolation from Plasma and NIPT Analysis

Genomic DNA was extracted using peripheral blood in EDTA according to the manufacturer’s instructions (MagCore Nucleic Acid Extraction Kit Diatech Pharmacogenetics, Jesi, Italy). For NIPT and clinical exome sequencing, a total of 10 mL peripheral blood was collected from the pregnant women in Streck blood collection tubes. The blood samples were first centrifuged at 1600× *g* for 10 min at 4°C to separate the plasma from peripheral blood cells. Cell-free DNA from 900 μL of maternal plasma was extracted using the QIAamp DNA Blood MiniKit (Qiagen, Hilden, Germany) following the manufacturer’s protocol. The pipeline for NIPT analysis included automated library preparation (VeriSeq NIPT Solution, Microlab STAR, Illumina, San Diego, CA, USA) and WGS sequencing on a Next550 (Illumina). The VeriSeq NIPT Assay Software (www.illumina.com/NIPTsoftware accessed on 6 November 2021) was used for data analysis of the aneuploidy status and fetal fraction from cffDNA. Details about NIPT analysis were reported previously [16].

### 2.3. DNA Extraction, PCR and Karyotype Analysis

Amniotic fluid was drawn, and genomic DNA was extracted from the amniocyte using the QIAamp DNA Blood Mini Kit (Qiagen). The quantitative fluorescent polymerase chain reaction test for rapid aneuploidy detection was performed using the Devyser Compactv3 QF-PCR Kit (QF-PCR;Devyser Compactv3, Devyser, Stockholm, Sweden) as previously described [17]. The amplified DNA samples were separated by electrophoresis using an ABI 3130xl Genetic Analyzer, and the analysis of each allele for specific markers was performed using GeneMapper Software ver. 4.0 (Applied Biosystems, Waltham, MA, USA). GTG-banding analysis of amniotic fluid was performed in established cell cultures following standard laboratory protocols. Forty metaphases were analyzed with the CytoVision software (CytoVision Version 7.6, Leica Biosystems, Richmond, IL, USA).

### 2.4. Target Region Capture Sequencing and Bioinformatics Analysis

Before starting with exome sequencing, cffDNA was amplified and repaired using the TruSeq Chip Sample Preparation Kit (Illumina Inc., San Diego, CA, USA), which includes End Repair, A-Tailing, Ligate Adapters, and Enrich DNA Fragments in order to obtain an adequate quantity to be used for DNA sequencing.

DNA quantification was performed using a Qubit 3.0 Fluorometer with the Qubit dsDNA HS (High Sensitivity) Assay Kit fluorescent dye method. Following the manufacturer instructions, we used 100 ng of DNA to perform library preparation (TruSight One kits, Illumina Inc., San Diego, CA, USA). After the target region sequence was captured and enriched, the resulting DNA libraries were quantified with the Qubit dsDNA HS Assay Kit fluorescent dye method to determine equimolar amounts for each library. Sequencing was carried out on NextSeq 500 (Illumina Inc., San Diego, CA, USA) to mean sequencing depth of at least 300×. Sequence data were aligned to the human reference genome GRCh37 (http://www.ncbi.nlm.nih.gov/projects/genome/assembly/grc/human/index.shtml, accessed on 1 February 2018) using the Burrows–Wheeler Aligner with default parameters. Trimming, base calling, coverage analysis, and variant calling were performed using an in house bioinformatic pipeline taking advantage of the following free software: bcl to fastq version 2.20, Isaac Aligner version 4, GATK “Genome Analysis Toolkit” version 4, samtools version 1.9, and bedtools version 2.

Vcf analysis was performed using the Illumina Variant Interpreter filtered by quality >15, by small variant consequences such as stop gains, sand losses, splice donors, splice acceptors, splice region, frameshift indels, in frame deletions, in frame insertions, initiator codon (ATG) losses, missense protein altering, incomplete terminal codon. Additional filtering was performed at frequency <0.05 in European populations using reference datasets such as the 1000 Genomes Project (https://www.internationalgenome.org/, accessed on 1 February 2018), gnomAD (https://gnomad.broadinstitute.org/, accessed on 10 February 2018), Exome Aggregation Consortium (http://exac.broadinstitute.org/, accessed on 10 February 2018), and Human Gene Mutation Database (HGMD) (http://www.hgmd.cf.ac.uk/ac/, accessed on 10 February 2018). A panel of sexual disease genes reported in Supplementary Table (Table S1) was used in order to search for a causative variant. This list was generated using online databases such as OMIM and HGMD professional using the keyword “sex.” Variants were classified according to the American College of Medical Genetics and Genomics (ACMG) guidelines [18]. We only selected variants affecting coding exons or canonical splice sites. Finally, synonymous variants were filtered out to detect only rare variants (frequency of <0.1%) in both dbSNP138 and our in-house database containing >1000 exomes) with high quality. This strategy was applied to both cffDNA and genomic WES.

### 2.5. Mutation Validation

Sanger sequencing was used to validate the mutation identified in amniotic fluid as well as in the heterozygous subjects by targeted sequencing. Primer3 (http://bioinfo.ut.ee/primer3-0.4.0/, accessed on 15 February 2018) was used to design primers at least 100 bp upstream and downstream from the mutation. Primer sequences are available on request. The genomic sequences from GenBank accession numbers NM_000197.2 were used as the reference sequence. PCR was performed to amplify the target fragments on a thermocycler instrument (SimpliAmp, ThermoFisher, Waltham, MA, USA), and the annealing temperature of PCR was 58 °C.DNA was amplified in a 50 µL reaction volume using AmpliTaq Gold 360 DNA polymerase (ThermoFisher), 0.2 µM each primer, 100 ng of genomic DNA. The amplified products were isolated by electrophoresis on 1.5% agarose gel and purified using the QIamp Purification Kit (Qiagen, Valencia, CA, USA). Sequence analysis was performed on an ABI Prism 3500 genetic analyzer (ThermoFisher).

## 3. Results

### 3.1. NIPT Analysis

NIPT analysis on the investigated pregnant woman did not detect trisomy in chromosomes 21, 18, and 13 with a DNA fetal fraction of 4%. The presence of a Y chromosome was also reported. During a follow up ultrasound examination of fetus, the external genitalia was consistent with a female. The cffDNA test was repeated to exclude a false positive with NIPT screening, and the results showed a XY male fetus again, with 8% of DNA fetal fraction. Due to this cffDNA-ultrasound sex discordance, the pregnant underwent an invasive prenatal diagnosis for additional investigations.

### 3.2. Cytogenetic Analysis

**Figure 1:**
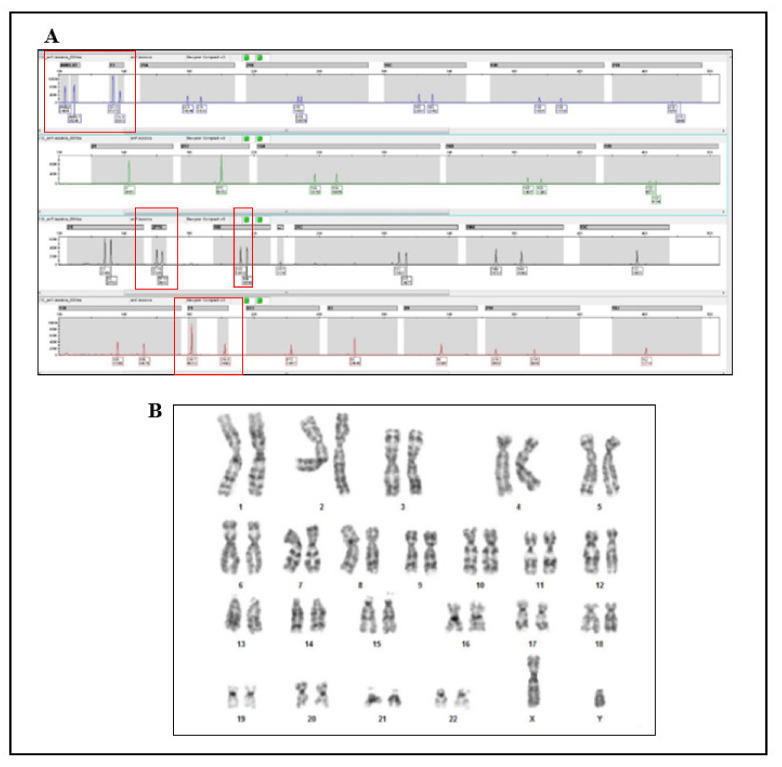
Conventional and molecular cytogenetics characterization on amniotic fluid of the fetus. (**A**) QF-PCR detected 46, XY. (**B**) GTG banding analysis of amniotic fluid showed a male normal karyotype.

QF-PCR on amniotic fluid showed a profile consistent with fetuses disomic for 13, 18, 21, and the presence of one X and one Y chromosome (SRY present), confirming the genotype described by NIPT (Figure 1A). In particular, all informative autosomal STR markers demonstrate a normal 1:1 marker ratio (Figure 1A). The presence of an informative X chromosomal STR marker confirms the dosage of one X chromosome. Fetal sex is assessed by the presence of the X chromosome-specific product of the AMXY, and the presence of Y-chromosome-specific product of the AMXY. T1 and T3 markers, which represent autosomal chromosomes, are in the ratio 2:1 with respect to X chromosomes. The SRY product was compatible with the presence of the Y chromosome. Chromosomal analysis using GTG-banding revealed a 46, XY karyotype (450 bands of resolution) (Figure 1B).

### 3.3. Molecular Findings

We performed trio-exome sequencing on cffDNA (plus maternal and paternal gDNA) first and on DNA from the amniotic fluid after.

The TruSight One Sequencing Panel provides comprehensive coverage of >4800 disease-associated genes. The probes on the panel covered about 4800 disease-associated genes. Using the targeted panel, a total of 712 variants were detected in cffDNA and 3461 variants in amniotic fluid, respectively.

**Figure 2:**
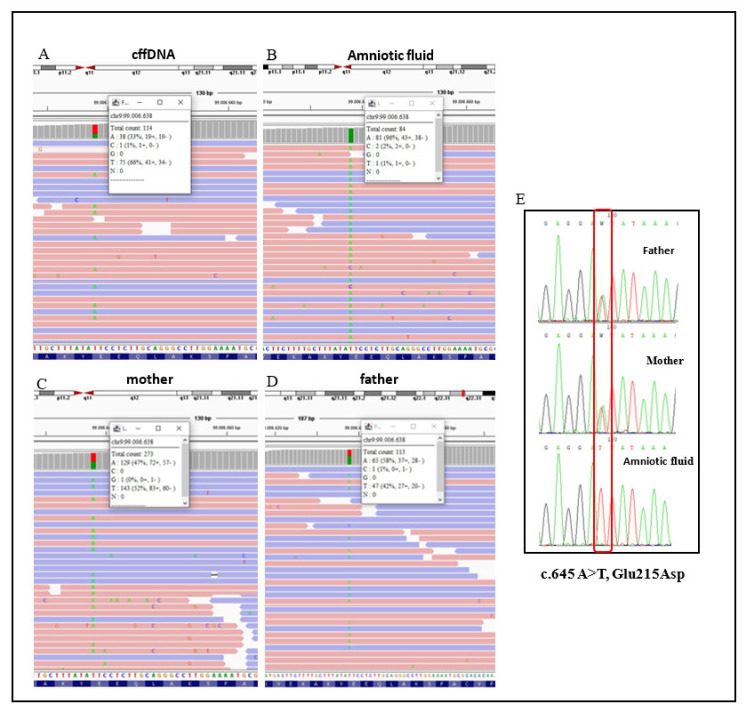
Molecular analysis of *HSD17B3*. (**A**–**D)** Photographs of Integrative Genomics View (IGV) representation of the c.645 A>T, p.(E215D) variant in cffDNA (**A**), amniotic fluid (**B**), and the parents (**C**,**D**). (**E**) Sanger sequencing data analyzed with Chromas. Electropherograms show that the missense variant c.645 A>T was detected in a homozygous state in the amniotic fluid and in a heterozygous state in the parents.

Both exome analysis identified a missense variant in the *HSD17B3* gene (Figure 2), which caused the substitution c.645 A>T in exon 9, and aminoacidic change glutamate to aspartate at position 215 of the protein. The variant was found at the heterozygous state in the cffDNA (with a reading frequency of ~33%) (Figure 2A) and the unaffected parents (Figure 2C,D), whereas genetic analysis revealed the presence of the homozygous E215D mutation in amniotic fluid (Figure 2B). No other gene mutations associated with DSD were present in the WES dataset. Sanger sequencing was performed on DNA extracted from amniotic fluid and peripheral blood of both parents. Results showed the c.645 A>T (p. Glu215Asp) mutation in a homozygous state in amniotic fluid DNA and in a heterozygous state in both parents’ DNA (Figure 2E).

**Figure 3:**
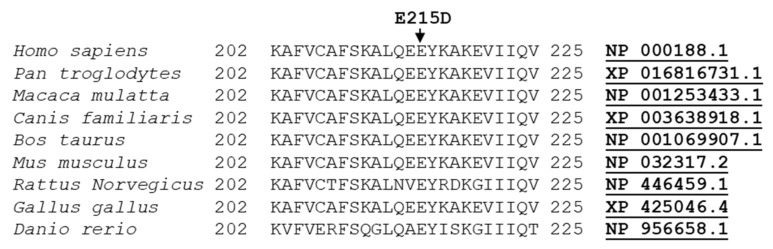
Alignment analysis of the amino acid sequences of *HSD17B3* from different species, showing complete conservation of the identified mutated residue (BLACK arrow).

The variant was predicted as probably damaging (Polyphen2) and damaging (Mutation Taster, SIFT), and was absent in the gnomAD database. In addition, the variant has been predicted as likely pathogenic (C4), according to the ACMG criteria (PS1, PM2, PP2, PP3, PP5). Sequence alignment of the region of *HSD17B3* protein bearing the pathogenic variant Glu215Asp across closely related species revealed a high degree of conservation (Figure 3).

## 4. Discussion

In children with disorders of sex development (DSD), a gender assignment is important for a correct case management such as for genital surgery and for social consequences. In our report, we describe a case of sex discrepancy between NIPT results and the ultrasound examination of the fetus during prenatal screening. For the first time, we used exome sequencing and non-invasive prenatal detection, providing an early diagnosis of 17β-HSD3 deficiency.

Among the 46, XY disorders of sex development (DSD) 17β-HSD3 deficiency is considered the most common form of testosterone biosynthetic defects [19]. It is very rare, at least in Western countries, whereas it is relatively frequent in the Arab population of the Gaza Strip [2,20]. According to a nation-wide survey in the Netherlands, the incidence of 17β-HSD3 deficiency is about 1:147,000 newborns [21]. The 17β-HSD3 enzyme is involved in the last step of testicular steroidogenesis, in which Δ4-androstenedione (Δ4-A) is converted into testosterone [1,22] and its defect results in undermasculinization characterized by hypoplastic-to-normal internal genitalia (epididymis, vas deferens, seminal vesicles, and ejaculatory ducts) but female external genitalia and the absence of a prostate [1,2,3]. Virilization occurs at puberty and is probably due to alternative 17β-HSD isoenzyme pathways or by the residual activity of the mutated 17β-HSD3 enzyme [23]. At this time, some children with 17-βHSD3 deficiency, usually reared as girl before puberty, adopt a male gender identity [1], whereas in several countries, they have almost female genitalia and are raised as girls and undergo gonadal surgery [4,21,23].

To date, there are 69 mutations identified in this gene (https://www. hgmd.cf.ac.uk/ac/gene.php?gene=*HSD17B3*, accessed on 2 October 2021). Mostly, these are missense and non-sense mutations, but splicing mutations as well as small or gross deletion/insertions have been described. In the present study, exome sequencing on both cffDNA and amniotic fluid revealed that they had the missense mutation c.645 A>T, E215D, which was confirmed by Sanger sequencing, inherited from the heterozygous parents (Figure 2). In the cffDNA, the E215D variant had a reading frequency of ~33% (Figure 2), therefore potentially excluding its presence in fetal DNA. However, because both parents were found to be heterozygous for the E215D mutation and the limited source material (DNA fetal fraction 8%), we decided to further evaluate this point, performing exome sequencing on DNA extracted from the amniotic fluid. Our variant is reported in dbSNP(https://www.ncbi.nlm.nih.gov/projects/SNP/snp_ref.cgi?searchType=adhoc_search$type=rs&rs=rs115063639, accessed on 2 October 2021) as a variant of uncertain clinically significance and in database HGMD as associated with pseudohermaphroditism. In silico predictions indicated the variant as likely pathogenic, according to ACMG classifications. The amino acid glutamate at this site is highly conserved in evolution (Figure 3) and was previously reported to inactivate the enzyme in vitro [23]. The E215D variant was previously described in patients who were raised as a female until puberty, when they were evaluated for virilization [23,24,25]. These patients presented female or ambiguous genitalia at birth, male behaviors, pubertal virilization, absence of menses, and male gender role [23,24,25]. They came from different countries, three were White Brazilian [23,24,25], while the other was English [24]. Our proband is the first case reported in Italy with this mutation. 17βHSD3 deficiency highlights that the variable phenotype manifests both clinically and biochemically before puberty, and occasionally in adult life. In fact, in genotypically identical cases, as it occurs within families, phenotypic variation for external sexual development was observed [23]. Furthermore, 17βHSD3 deficiency in prepubertal patients is often clinically indistinguishable from androgen insensitivity syndrome (AIS), particularly in the case of severe undervirilization of the external genitalia in *HSD17B3*, causing difficulties in accurate diagnosis [2]. Usually, the differential diagnosis is made by demonstrating a low serum testosterone/androstenedione (T/A) ratio (<0.8) following a short hCG stimulation test [24]. However, the endocrine evaluation of patients with 46, XY DSD is not reliable in all cases, as reported previously [4,26]. For instance, Bertelloni et al. described very young infants (less than six months old) with a lower sensitivity (89%) of the stimulated T/Δ4A ratio [4]. Indeed, molecular analysis for responsible genes is mandatory not only for accurate diagnosis and genetic counselling, but also for the decision regarding gender assignment. Ideally, these patients should be genetically diagnosed as newborns or prenatally in order to enable thorough consideration of the different factors affecting the two different alternatives as early as possible, so that an optimal decision can be made. In 2009, the first case of prenatally identified 17β-HSD3 deficiency was reported in a child with discordance between the 46, XY karyotype and female external genitalia with phallic structure [4].

In our case, we suspected a sex development disorder during prenatal age based on sex discordance between NIPT and ultrasound screening. NIPT analysis showed the presence of Y chromosome confirmed by prenatal chromosomal analysis, which showed a male with a normal karyotype (46, XY), whereas at ultrasound screening, female external genitalia were evident. Through exome sequencing (ES) on cffDNA and parents, we first identified the heterozygous mutation E215D in *HSD17B3* gene as a possible cause of sex discrepancy in our patient. Then, the confirmation of the molecular diagnosis on DNA extracted from the amniotic fluid (on which the E215D was found in homozygous) allowed us to provide a correct and rapid diagnosis of 17β-HSD3 deficiency. A large number of genes are involved in testicle development and testosterone production and action, and their mutations may lead to 46, XY DSD. The diseases are genetically heterogeneous and have variable clinical features. Recently, it has been shown that NGS is becoming the preferred approach for a definitive non-invasive prenatal diagnosis (NIPD) of single gene disorders at an early gestational stage without the need for invasive testing [27]. Techniques of exome sequencing allowed us to reveal the presence of monogenic diseases after an invasive prenatal diagnosis showed a normal karyotype in fetuses with abnormalities at ultrasound [28,29]. The use of targeted NGS panels or WES for molecular diagnosis of 46, XY DSD patients has already been reported successfully in several previous publications with a higher rate of correct diagnosis and reduced diagnostic delay in comparison with single gene sequencing [30,31]. Hence, all genes with any involvement in sex development can be analyzed concurrently, and with WES, new genes can be included in the analysis not currently available for clinical testing.

In our proband, we performed clinical exome sequencing (approximately 4800 disease-associated genes) focusing subsequently on a specific subset of genes involved in 46, XY DSD with a rapid diagnosis of 17β-HSD3 deficiency (Table S1). In particular, the present case demonstrated the clinical utility of cffDNA based screening in the differential diagnosis of cases whose NIPT results for sex chromosome aneuploidy are discordant with ultrasound screening or fetal karyotyping.

The limited amount of fetal cffDNA and its heavy dilution by maternal cffDNA present significant technical challenges. In addition, NIPD for monogenic disorders is less widely used elsewhere in the world, where it is largely delivered on a research basis and for high-risk pregnancies [10]. We tried to overcome these limitations by performing prenatal ES of parent–fetus trios using DNA extracted from amniotic fluid and therefore, the fetal risk for monogenic diseases was assessed based on the carrier status of the biological parents. In summary, the introduction of cffDNA testing into prenatal screening has made sex chromosome assessment possible from 10 weeks of gestation and discordant fetal sex could be more commonly diagnosed prenatally. Thus, correct diagnosis in sex development disorders should be made early so that treatment, management, and genetic counseling can be specifically directed.

## 5. Conclusions

The diagnosis of 46, XY DSD is very challenging and not rarely is confirmed only at older ages, when an affected XY female presents with primary amenorrhea or develops progressive virilization. This can be overcome with sequencing of a group of genes or whole exome. Genetic diagnoses are useful for patients and clinicians, contribute to clinical knowledge of DSD, and are invaluable for genetic counseling of couples contemplating future pregnancies. The present case demonstrates the clinical utility of cffDNA based screening in the differential diagnosis of cases whose NIPT results for sex chromosome aneuploidy are discordant with ultrasound screening or fetal karyotyping. Discordance between NIPT gender results and the phenotypic features requires further prenatal and postnatal investigation of the fetus.

## Data Availability

All data generated or analyzed during this study are included in this published article (and its Supplementary Materials). Protocols and deidentified, aggregated data that underlie the results reported in this article are available for non-commercial scientific purposes upon reasonable request from the corresponding author. For privacy reasons, the raw data are not publicly available.

## Supplementary Materials

The following are available online at https://www.mdpi.com/article/10.3390/genes12121890/s1, Table S1: Gene included in the DSD Panel.
