# Supplementary material for: Detection of 46, XY Disorder of Sex Development (DSD) Based on Plasma Cell-Free DNA and Targeted Next-Generation Sequencing
Source: Genes (Basel). 2021 Nov 25;12(12):1890. doi: 10.3390/genes12121890 (PMC8700836; doi:10.3390/genes12121890)
Supplement: Supplementary file 1 [file genes-12-01890-s001.zip › genes-1477055-supplementary.pdf]

Table S1: Gene included in the DSD Panel.

**SEX DETERMINATION (gonadal development, complete or partial gonadal disgenesis)**				
**Gene**	**Locus**	**OMIM**	**Associated DSD**	**Inheritance**
BMP15	Xp11.22	300247	46, XX DSD—ovarian dysgenesis	AD
CBX2	17q25.3	602770	46, XY DSD CGD	AR
DHH	12q13.12	605423	46XY PGD or CGD	AR, AD
DMRT1	9p24.3	602424	46, XY DSD	AD: deletion
DMRT2	9p24.3	604935	46, XY DSD	AD: deletion
FOXL2	3q22.3	608996	POI alone or with blepharophimosis, ptosis, and epicanthus inversus syndrome	AD
GATA4	8p23.1	600576	46, XY DSD	AD
NR0B1	Xp21.2	300473	46, XY GD—gain of function; 46, XX CAH with HH	XL-dup or XLR
NR5A1	9q33.3	184757	46, XY DSD (various); 46, XX POI	AD
MAP3K1	5q11.2	600982	46, XY GD	AD
RSPO1	1p34.3	609595	46, XX OT DSD with palmoplantar hyperkeratosis	AR
SOX3	Xq27.1	313430	46, XX T or OT DSD—gain of function	XL: dup
SOX9	17q24.3	608106	46, XY GD and campomelic dysplasia; 46, XX T DSD—duplication	AD
SRY	Yp11.2	480000	46, XX T DSD—gain of function; 46, XY ovarian DSD	Translocation
TSPYL1	6q22.1	604714	46, XY DSD with sudden infant death syndrome	AR
WNT4	1p36.12	603490	46, XY ovo or OT DSD or 46, XY CGD—duplication; 46, XX T DSD; 46, XX MRKH	AD: dup; AR; AD
WT1	11p13	607102	Frasier syndrome and Denys-Drash	AD
ZFPM2	8q23.1	603693	46, XY GD	AD
**SEX DIFFERENTIATION (androgen synthesis and action)**				
**Gene**	**Locus**	**OMIM**	**Associated DSD**	**Inheritance**
AKR1C2	10p15.1	600450	46, XY DSD	AR
AKR1C4	10p15.1	600451	46, XY DSD	AR
AMH	19p13.3	600957	PMDS	AR

AMHR2	12q13.13	600956	PMDS	AR
AR	Xq12	313700	46, XY DSD. Complete AIS/partial AIS, isolated hypospadia	XL
ARX	Xp21.3	300215	X-linked lissencephaly with ambiguous genitalia	XL
ATRX	Xq21.1	300032	46, XY DSD associated with alpha-thalassemia X-linked intellectual disability syndrome	XL
CDKN1C	11p15.4	600856	Genital anomalies in association with Beckwith-Wiedemann and IMAGE syndrome	AD
CYB5A	18q22.3	613218	46, XY DSD	AR
CYP11A1	15q24.1	118485	46, XY sex reversal (partial or complete) with adrenal insufficiency. CAH	AR
			Hypospadias	AD
CYP11B1	8q24.3	610613	46, XX DSD. CAH due to steroid 11-beta-hydroxylase deficiency	AR
CYP17A1	10q24.32	609300	46, XY DSD. 17,20-lyase deficiency CAH	AR
CYP19A1	15q21.2	107910	46, XY DSD. Aromatase deficiency	AR
CYP21A2	6p21.33	613815	46, XX DSD virilization—21-hydroxylase-deficient CAH	AR
FGFR2	10q26.13	176943	46, XY GD with craniosynotosis. Apert syndrome	AD
HSD17B3	9q22.32	605573	46, XY DSD—17-β-hydroxysteroid dehydrogenase III deficiency	AR
HSD17B4	5q23.1	233400	Perrault syndrome (with ovarian dygeneisis in 46, XX)	AR
HSD3B2	1p12	613890	46, XY DSD and 46, XX DSD—3-β-hydroxysteroid dehydrogenase-deficient CAH;	AR
LHCGR	2p16.3	152790	46, XY DSD—Leydig cell hypoplasia,	AR
			Precocious puberty (male)	AD
NR3C1	5q31.3	138040	46, XX hyperandrogenism	AD

POR	7q11.23	124015	Cytochrome P450 oxidoreductase deficiency	AR
SRD5A2	2p23.1	607306	46, XY DSD. Steroid 5-α-reductase deficiency	AR
			Hypospadias	AD
STAR	8p11.23	600617	46, XY DSD—cholesterol desmolase-defient CAH	AR
**Central causes of hypogonadism**				
**Gene**	**Locus**	**OMIM**	**Associated DSD**	**Inheritance**
BBS9	7p14.3	615986	Bardet-Biedl syndrome	AR
CHD7	8q12.2	608892	CHH or KS. CHARGE syndrome	AD
FGF8	10q24.32	612702	CHH or KS	AD
FGFR1	8p11.23	147950	CHH or KS	AD
FSHB	11p14.1	136530	CHH	AD
FSHR	2p16.3	136435	46, XX ovarian dysgenesis	AR
GNRH1	8p21.2	152760	CHH	AR
GNRHR	4q13.2	138850	CHH	AR
HESX1	3p14.3	601802	KS or CPHD	AD
KAL1	Xp22.31	300836	CHH or KS	XL
KISS1R	19p13.3	604161	CHH or KS	AD
LEP	7q32.1	164160	CHH with obesity	AR
LHX3	9q34.3	600577	CPHD	AR
PROK2	3p13	607002	CHH or KS	AD
PROKR2	20p12.3	607123	CHH or KS	AD
PROP1	5q35.3	601538	CPHD	AR
TAC3	12q13.3	162330	CHH	AR
WDR11	10q26.12	606417	CHH or KS	AD
**Other (isolated hypospadia, cryptorchidism, MRKH):**				
**Gene**	**Locus**	**OMIM**	**Associated DSD**	**Inheritance**
ATF3	1q32.3	603148	46, XY isolated hypospadias	
HOXA13	7p15.2	142959	Hand-foot uterus syndrome - MRKH in 46, XX	AD
			Guttmacher syndrome in 46, XY including hypospadias	AD
INSL3	19p13.11	146738	Cryptorchidism	AD
MAMLD1	Xq28	300120	Hypospadias	XLR
RXFP2	13q13.1	606655	Cryptorchidism	-
